# Robotic-Assisted Nephrectomy for Living Kidney Donation—Single Center Initial Experience (Case Series) and Review of the Literature

**DOI:** 10.3390/jcm13133754

**Published:** 2024-06-27

**Authors:** Karolina Kędzierska-Kapuza, Inga Łopuszyńska, Agnieszka Mizerska, Marta Matejak-Górska, Krzysztof Safranow, Marek Durlik

**Affiliations:** 1 Department of Gastroenterological Surgery and Transplantology, National Medical Institute, Ministry of Interior Affairs and Administration, Wołoska St. 137, 02-507 Warsaw, Poland; 2 Department of Biochemistry and Medical Chemistry, Pomeranian Medical University in Szczecin, Powstańców Wlkp. 72, 70-111 Szczecin, Poland

**Keywords:** robotic-assisted nephrectomy, kidney transplantation, living donor, RANLD

## Abstract

**Background**: Robotic-assisted nephrectomy for living kidney-donation (RANLD) has the potential of becoming the leading technique of harvesting kidney, if expertise is available. The aim of this work is to present our initial experience with robotic technique with additional hand-assistance. **Materials and Methods**: We initiated RANLD at our clinic using the DaVinci System in September 2022, since then harvesting six kidneys, four left and two right; in two cases, multiple arteries existed. The renal vessels were ligated using vascular staplers. All the operations included a hand-assist with the use of Gelport. The mean operation time was 119.2 min (SD 12 min). **Results**: There were no conversions or donors’ post-operative complications. Time of discharge from the hospital was 4.5 days post-operatively. Total hospital length of stay was 7.8 days. All the harvested kidneys were transplanted, five of them with adequate function, three with initially delayed function, and one needed to be removed due to thrombotic complications. Post-operative was pain assessed on the VAS scale and overall pain was assessed according to the NRS scale. At the discharge day, donors’ performance status was about 87.5% according to the Karnofsky scale. The donors resumed their normal life activity within 15.7 days and returned to work within 45.2 days. The serum mean creatinine level before the donation was 0.85 mg/dL (SD 0.1 mg/dL), and mean eGFR (MDRD) = 91.8 mL/min/1.73 m^2^ (SD 16.1 mL/min/1.73 m^2^). **Conclusions**: Further development of RANLD could lead to an increase in the number of living kidney donors, particularly in Poland where the number is currently lower than that of deceased donors. Prolonged operation time, longer warm ischemic time, and high equipment costs are significant drawbacks of RANLD.

## 1. Introduction

Laparoscopic donor nephrectomy (LDN), firstly introduced in 1995, has become the gold standard technique for living kidney donation ever since [1]. Thanks to the reduction in post-operative pain, a shorter length of hospital stay and better cosmetic results, this approach offers important advantages and results in wider acceptance by patients. Multiple variations have already been introduced, including not only an intra-abdominal approach, but also a retroperitoneal, either with or without hand-assistance [2]. In any technique, safety and convenience for the donor must remain crucial. As efficient as they are, laparoscopic techniques still have their limitations, such as the 2-D imaging, limited instrument motion and surgeon’s discomfort. The growing availability and popularity of robotic techniques in surgery implicate their application in living kidney donation programs, therefore posing a question regarding their potential benefits in comparison with the other approaches.

There is already some evidence of the advantages of robotic techniques, such as a preferable safety profile for the donor and higher repetitiveness of the results [3].

In this paper, we present our initial experience with robotic-assisted nephrectomy for a living kidney-donation (RANLD) as a series of six cases, performed between September 2022 and January 2024, using the surgical robotic system DaVinci Xi.

## 2. Materials and Methods

For donor nephrectomy, we use an anterior, transperitoneal approach. The patient is lying with his side elevated to 90 degrees, according to the side of the nephrectomy. The pneumoperitoneum is created by using the Veress needle, preferably at the Palmer’s point, and the gas pressure is maintained at the 15 mm Hg level. The port for the oblique optic robotic camera is placed in the mid-clavicular line next to the umbilicus, and then three other ports for the robotic instruments are placed at an equal distance from each other in line (Da Vinci Xi System). The robotic patient cart is located on the ipsilateral side and approaches the patient from behind. After setting the anatomical target and docking all robotic arms, the instruments are advanced—usually Cadiere forceps, fenestrated bipolar forceps, and monopolar cautery hook. Apart from these robotic instruments, we also use two ports for surgical assistance—one 10 mm port located in the upper midline and the other, the Gel-Port System, introduced through one-side by Pfannenstiel incision, used both for hand-assistance and for the organ extraction. The peritoneal wall is incised at the side and, with the colon being gently retracted, access to the retroperitoneal space is gained. Care must be taken to avoid any duodenal or pancreatic damage. Mobilization of the kidney proceeds from its dorsal side by incising its adipose sac. The ureter has to be localized before its crossing of the iliac vessels, its distal end clipped by Hem-o-Lok, cut and then advancing proximally, carefully excised with an adequate portion of the surrounding tissues to avoid post-transplant ischemia. The Da-Vinci EndoWrist technology with its improved dexterity enables careful dissection of vessels at the origin, making them long enough for a convenient transplantation, which is a special challenge in the case of the right renal vein. The artery can be safely secured by the use of staplers. For the vein closure, a vascular endo-stapler might be used. The harvested kidney is then removed from the abdomen by the surgical assistant through the pre-existent incision, therefore minimizing the warm ischemic time, immediately put in ice and, from this point on, prepared by the transplanting surgical team on the “back-table”. A meticulous check om hemostasis and placement of a drain in the lodge of the removed kidney, with subsequent undocking of the robot and closure of the incisions, finish the procedure.

## 3. Results

As presented in Table 1, in our group of harvested organs, two were right kidneys and four left, and in two cases additional renal vessels existed. Renal vascular anatomy was assessed by spiral computed tomography with a 3-D vessel reconstruction. The side of nephrectomy was chosen according to the more favorable vascular anatomy, the left side being preferred.

The operation time was 105–135 min, regardless of the side of the operation and with significant reduction of the operation time from first to last procedure. There was no conversion to the open procedure.

**Figure 1:**
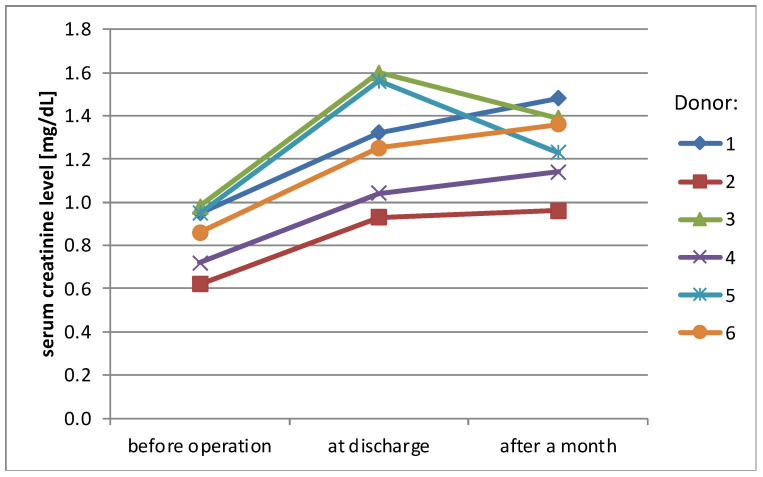
Serum creatinine levels of donors.

**Figure 2:**
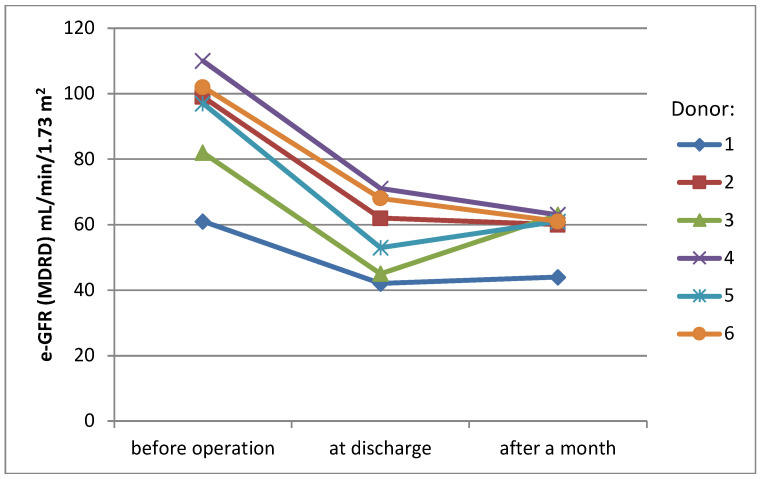
eGFR values of donors.

Mean serum creatinine level before surgery was 0.85 mg/dL (SD ± 0.1 mg/dL), rising to 1.28 mg/dL (SD ± 0.3 mg/dL) at the day of discharge (38% rise) and stabilizing at the level 1.26 mg/dL (SD ± 0.2 mg/dL) one month later (36% rise, compared to the initial level) (Figure 1). eGFR (MDRD) presented a similar pattern of changes over time—mean value before surgery was 91.8 mL/min/1.73 m^2^ (SD ± 16.1 mL/min/1.73 m^2^), 56.8 mL/min/1.73 m^2^ (SD ± 11 mL/min/1.73 m^2^) at the day of discharge and 58.7 mL/min/1.73 m^2^ (SD ± 6.6 mL/min/1.73 m^2^) one month later (change −38% and −36%)(Figure 2).

WIT1, also called donor warm ischemia time, was the time from clamping of the aorta or renal artery to cold perfusion. In our cohort, the WIT1 mean value was 9 min (SD ± 4.3 min), showing improvement via the learning curve (see Table 1)

The mean hospital length of stay of the donors was 7.8 days (SD ± 1 day). All harvested organs were transplanted, five of them functioning, with transitional delayed graft function in three patients. One kidney needed to be removed due to venous thrombotic complications. All donors were asked to assess their performance status according to the Karnofsky scale, with a mean result of 87.5% (SD ± 6%). The mean time to resume normal life activity was 15.7 days (SD ± 4 days), returning to work within a mean 45.2 days (SD ± 23 days).

**Figure 3:**
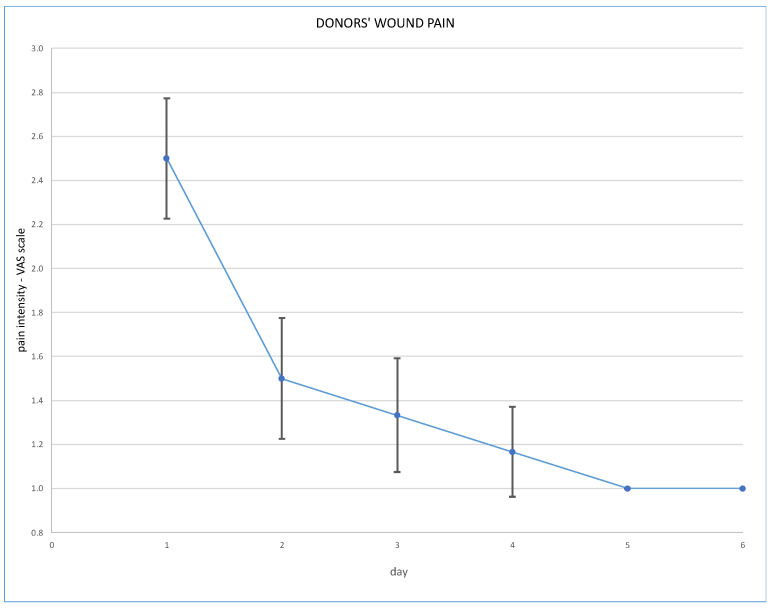
Post-operative wound pain at VAS scale.

**Figure 4:**
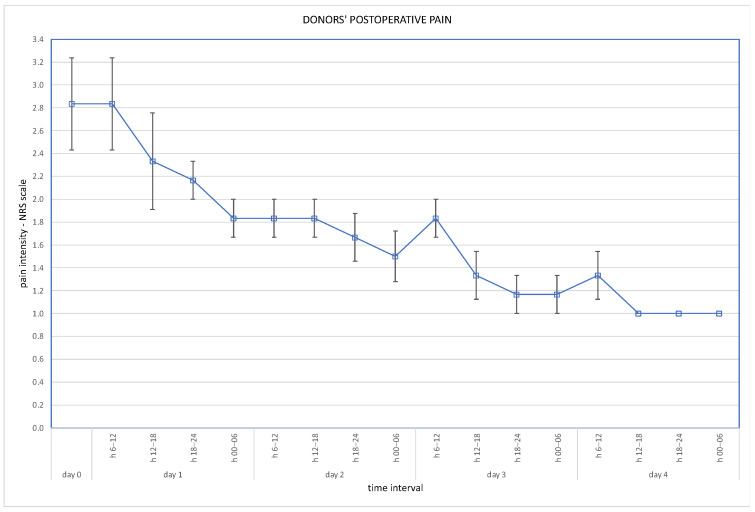
Overall pain at NRS scale (days 1–4 assessed in 6-h intervals).

Post-operative wound pain was assessed using the Visual Analogue Scale (VAS), and overall pain via the Numeric Rating Scale (NRS); results are presented in Figure 3 and Figure 4.

Post-operative analgesics were used for 2–5 days, consisting of total oxycodone mean dose 0.03 g iv (SD ± 0.01 g), total paracetamol mean dose 6.6 g iv (SD ± 2.3 g), and total metamizole mean dose 2.5 g iv (SD ± 1.1 g).

Time of performing anastomoses (recipient warm ischemia time, WIT2) varied depending on anatomical conditions. WIT2 in our cohort was 20–25 min (mean 22.5 min, SD ± 3 min). The mean cold ischemia time in our cohort (CIT) was 82 min (SD ± 44). Data concerning recipients are presented below (Table 2).

The circumstances connected with recipient 4 graft loss were as follows: while preparing the kidney on a back table, it was noticed that small perihilar vessels were damaged, which required repair, therefore CIT was 135 min. Immediately after transplantation, Doppler ultrasound was performed and thrombosis of artery and vein were discovered. The patient returned to the operation theater. Re-operation was performed, and thrombus removed from both vessels. Despite this immediate action, there was no blood flow through the artery and vein of the graft (an intra-operative Doppler ultrasound examination was performed). After reconciliation, at this time graftectomy was performed. Histopathology examination showed: “kidney with normal structure, congested. Apart from the features of discrete fading of vascularization, no other abnormalities were detected. In the lumen of tubules, single microcalcification was found”.

## 4. Discussion

The main concern during qualification for a living kidney donation is the safety of the donor. It is crucial that kidney harvesting cannot lead to any health status deterioration, neither perioperatively nor in long-term follow-up. It is also achieved by a system of long-term care designed for organ donors. Thanks to regular and careful health evaluation, it is possible to diagnose and treat any potential pathologies at an early stage, thus reducing the risk of further complications.

The robotic-assisted surgery offers potential advantages over the traditional laparoscopy—the improved dexterity correlated with the EndoWrist technology of the instruments and better visualization with the high resolution 3-dimension camera.

There is evidence that, with growing experience of the surgical team, the procedure can be performed safely without any hand-assistance [4,5]. Hand-assistance, albeit it might be useful if a major bleeding occurs and for a quick recovery of the kidney from the abdomen, is also a risk factor for wound complication. The body mass and posture of the donor might cause additional difficulties for the assist [6].

Robotic techniques proved to be beneficial for the donors’ post-operative quality of life, in terms of lower post-operative pain and less requirement for analgesics [7]. With regard to our cohort, post-operative pain assessed on the VAS scale immediately after the operation was 2.5 pts (SD ± 0.5 pts). On the second day it was 1.5 pts (SD ± 0.5 points), on the third day 1.3 pts (SD ± 0.5) and on the fourth day 1.2 pts (SD ± 0.4). Overall pain assessed on the NRS scale was a mean 2.5 pts on the day of operation, and 1.2 pts on the fourth day. Our routine practice of post-operative analgesics consists of oxycodone, intravenously or subcutaneously, for 24 h and paracetamol, or rarely metamizole, intravenously during the following days. Evidence is scarce supporting the use of particular analgesics for these patients. The analysis of Manne et al. concludes that intravenous paracetamol is less effective as an analgesic after kidney donation than tramadol [8].

Another potential advantage of the robotic techniques is a faster learning curve [5,9]. Given the high incidence of vascular abnormalities regarding renal vessels, including both arteries and veins, another possible technical difficulty arises, with robotic techniques being potentially advantageous [10]. Furthermore, preserving sufficient vascular length is of the utmost importance for safe and efficient anastomosis in the recipient, and the robotic approach might be beneficial, especially on the right side [7]. The improved dexterity of robotic techniques might also result in more accurate tissue preparation, leading to more extensive preservation of the periureteric tissues [9].

Application of robotic techniques is still limited by their high costs and selective availability for surgeons. High costs include both the robotic platform and all necessary instruments, designed with restricted times of possible use. It is also required that the surgeons are additionally trained and certified in order to use the platform. However, when discussing the economic aspects of the procedure, the reduced hospital stay and lower overall morbidity of the donors, as well as their post-operative quality of life and quicker return to normal activities, also have to be taken into account [11].

On the other hand, possible postoperative complications have to be considered. Chyle leaks post-nephrectomy, occurring in 0 to 1.8% of laparoscopic cases, pose the risk of malnutrition and a prolonged hospital stay. Limited data exists on robotic nephrectomies. Alwatari et al. reported two cases of robotic LDN with chyle leaks. One patient, despite no intra-operative leak, later required paracentesis due to abdominal symptoms, recovering well one-year post-donation. Another patient had a lymphatic leak during surgery but showed no signs of any chyle leak three months later, maintaining good renal function. The management includes dietary adjustments, nutrition support, and interventions, like paracentesis or duct ligation. Prevention strategies. like careful vessel sealing and low-fat diet, are crucial. Identifying risk factors, such as low BMI and multiple renal vessels, aids in the early detection and management for better outcomes [12].

Kawan et al. expressed reservations about RLDN, citing longer operating times and higher costs without any significant reported advantages [13]. Choosing between laparoscopic and robotic techniques for a donor nephrectomy is critical due to the procedure’s complexity. Both methods aim to minimize complications to protect the donor, the graft and the recipient. Concerns about laparoscopic donor nephrectomy’s impact on the graft function and the warm ischemia time have been debunked. While both the laparoscopic and robotic approaches can prepare the kidney and the vessels, current robotics offer no clear benefits over laparoscopy. Hand-assisted salvage during the laparoscopy ensures controlled conditions and immediate recovery, contrasting with the technical challenges and prolonged recovery times of robotic-assisted donor nephrectomy. Combining laparoscopic donor nephrectomy with parallel robotic situs preparation and renal vessel exposure appears to be the optimal approach for safe and cost-effective donor nephrectomy and subsequent robotic kidney transplantation. This combination marks the first exclusive use of the minimally invasive methods throughout the living donation process, from organ removal to transplantation.

Surgeons often worry about potential longer warm ischemia times. Windisch et al. compared RDN (robotic donor nephrectomy) to the more common hand-assisted laparoscopic nephrectomy (HLDN), focusing on warm ischemia time, total operative time, learning curve, hospital stay, donor renal function, and post-operative complications [14]. In a retrospective study of 176 cases, RDN and HLDN were compared. RDN favored left-sided nephrectomy (82% vs. 52%, *p* < 0.01) and had a longer operative time (287 vs. 160 min; *p* < 0.01), but similar warm ischemia time (221 vs. 213 s, *p* = 0.446). RDN resulted in a shorter hospital stay (3.9 vs. 5.7 days, *p* < 0.01) and slightly better renal function at the 1-month checkup (1.56 vs. 1.44, *p* < 0.01). Overall, RDN showed safety and efficiency compared to HLDN, with comparable warm ischemia times, despite longer operative durations. Monitoring donor renal function over an extended period is recommended for both techniques. [14]

The Swiss national database indicated a 27% rise in patients awaiting renal transplantation from 2010 to 2023, with live donor transplants constituting 34% during this period (1530 vs. 2994) [15]. Donation criteria have expanded to include older patients and those with relative contra-indications, like obesity, prediabetes, kidney stones, or hypertension, ensuring donor safety and quality of life similar to that of non-donors [16,17]. Minimally invasive techniques for living donor nephrectomy (LDN) are predominantly laparoscopic (57.4%) or hand-assisted (25.3%), with robot-assisted donor nephrectomy (RDN) representing only 1.3% of cases. Recent American research on 1084 patients, including a significant percentage of obese (39.4%) and overweight patients (34.1%), demonstrated the safety of RDN, even for those with higher BMI [18].

Broe et al. concluded in their systematic review that there is no significant difference between HALDN and pure LDN in perioperative outcomes [19]. Both techniques show similar rates of conversion to an open procedure and perioperative complications. HALDN consistently exhibits a shorter warm ischemia time (WIT) and procedure duration. However, higher-quality studies are necessary to definitively establish the superiority of one technique over the other. Limited statistical evidence does not favor either technique.

Creta et al. conducted a review covering 18 studies involving 910 patients undergoing RALDN from 2000 to 2018 [20]. The studies encompassed various designs: 44.4% retrospective observational, 22.2% prospective observational, 5.5% randomized controlled trial, and 27.7% case reports. Notably, 44.4% of the studies included a control arm. Findings revealed a wide range of mean operative time (OT) and mean console time (CT), from 139 to 306 min and 82 to 120 min, respectively. Some studies reported longer OT in RALDN compared to LDN, while others found similar outcomes. Warm ischemia time (WIT) varied from <1.5 to 5.8 min, with its impact on surgical technique and vascular anatomy remaining controversial. Intra-operative complications occurred in 0% to 6.7% of cases, with bleeding being the most common.

In our cohort, WIT1, also called donor warm ischemia time, mean value was 9 min (SD ± 4.3 min), showing improvement via the learning curve. Last donors’ WIT1 time lasted 2 min. We observed the donor’s postoperative stay to last 4–6 days, with no conversions to laparotomy. Creatinine levels on discharge day increased by a mean 38%, then and stabilized after a month, being a mean 36% higher than the initial value.

In the above cited publication, conversion rates (CR) ranged from 0% to 5%, and hospital stays (LOS) lasted 1 to 5.8 days. Postoperative pain was lower after RALDN compared to LDN in three studies. Late outcomes, like serum creatinine, eGFR, and ESRD, showed no significant differences between RALDN and LDN across studies.

Overall, evidence supports the safety of RALDN. Although some studies initially reported longer OT and WIT compared to LDN, improvements occurred with experience. RALDN showed potential benefits in blood loss, hospital stay, and pain compared to LDN, with comparable graft and recipient outcomes. However, caution is needed in interpreting these findings, and further research is necessary on RALDN’s role.

A 2023 meta-analysis compared robotic and laparoscopic donor nephrectomy across 12 studies (971 patients) [21]. It found no significant differences in operative time or blood loss. However, warm ischemia times were longer with robotic surgeries. Robotic procedures were associated with reduced postoperative length of stay and pain compared to laparoscopic methods. Complication and conversion rates were similar between the two approaches. Overall, robotic-assisted laparoscopic techniques offer limited additional benefits in donor nephrectomy, but further research is needed for validation.

## 5. Conclusions

Summary and key points from our study:

RANLD Potential: RANLD (Robotic assisted nephrectomy for living kidney donation) shows promise as a surgical technique for living kidney donation.

Increasing Living Donors: Further development of RANLD could lead to an increase in the number of living kidney donors, particularly in Poland, where the number is currently lower than that of deceased donors.

Technological Advantages: Robotic instruments offer opportunities to overcome limitations of minimally-invasive approaches, potentially reducing surgical complications and improving post-operative quality of life and safety for donors.

Disadvantages: Prolonged operation time, longer warm ischemic time, and high equipment costs are significant drawbacks of RANLD.

Shorter Learning Curve: Robotic techniques have a shorter learning curve compared to traditional laparoscopy, which facilitates ongoing development and refinement of RANLD.

Research Needed: Further research is required to address the disadvantages and improve the technique.

## Figures and Tables

**Table 1:** Donors’ characteristics.

Patient	Sex	Age	Comorbidities	Blood Type	HLA Mismatch	Side of Nephrectomy	Number of Renal Vessels	Time of Operation (min)	WIT1 (min)	Donor’s Complications	Length of Hospital Stay (Days)	Post-Operative Hospital Stay (Days)	The Karnofsky Performance Scale Index(%)	Return to Normal Activity (Days)	Return to Work (Days)
1	female	54	arterial hypertension, dyslipidemia, hyperuricemia	A+	4/6	left	1 artery, 1 vein	135	10	-	9	6	80	21	60
2	female	57	-	O+	2/6	right	2 arteries, 2 veins	135	11	-	7	5	95	14	30
3	male	47	dyslipidemia	A−	2/6	left	1 artery, 1 vein	120	11	-	8	4	90	10	21
4	female	38	-	A+	0/6	right	1 artery, 1 vein	105	14	Post-operative haernia month 5	8	4	80	21	90
5	male	52	overweight, dyslipidemia	O+	2/6	left	1 artery, 1 vein	110	6	-	8	4	90	14	40
6	male	57	dyslipidemia, ischaemic herat disease	O+	2/6	left	2 arteries, 2 veins	110	2	-	7	4	90	14	30

**Table 2:** Recipients’ data.

Recipient	CIT min	WIT2 min	Complications
1	40	20	none
2	63	25	DGF, 2 × HD, SARS-CoV-2 infection
3	44	20	DGF, 3 × HD
4	135	20	Thrombosis of vein and artery, immediate re-operation, graftectomy at day 0.
5	60	25	Transient bleeding from urinary bladder Day 7, no HD
6	150	25	DGF, 7 × HD

DGF—delayed graft function, CIT cold ischemia time, WIT2—warm ischemia time = time of anastomosis. HD hemodialysis.

## Data Availability

The original contributions presented in the study are included in the article, and further inquiries can be directed to the corresponding author.
